# Fibular flap for mandible reconstruction in osteoradionecrosis of the jaw: selection criteria of fibula flap

**DOI:** 10.1186/s40902-016-0093-x

**Published:** 2016-11-25

**Authors:** Ji-Wan Kim, Jong-Hyun Hwang, Kang-Min Ahn

**Affiliations:** Department of Oral and Maxillofacial Surgery, College of Medicine, University of Ulsan, Asan Medical Center, Ulsan, Republic of Korea

**Keywords:** Osteoradionecrosis, Fibular, Mandible, Free flap, Radiation, Oral neoplasm

## Abstract

**Background:**

Osteoradionecrosis is the most dreadful complication after head and neck irradiation. Orocutaneous fistula makes patients difficult to eat food. Fibular free flap is the choice of the flap for mandibular reconstruction. Osteocutaneous flap can reconstruct both hard and soft tissues simultaneously. This study was to investigate the success rate and results of the free fibular flap for osteoradionecrosis of the mandible and which side of the flap should be harvested for better reconstruction.

**Methods:**

A total of eight consecutive patients who underwent fibula reconstruction due to jaw necrosis from March 2008 to December 2015 were included in this study. Patients were classified according to stages, primary sites, radiation dose, survival, and quality of life.

**Results:**

Five male and three female patients underwent operation. The mean age of the patients was 60.1 years old. Two male patients died of recurred disease of oral squamous cell carcinoma. The mean dose of radiation was 70.5 Gy. All fibular free flaps were survived. Five patients could eat normal diet after operation; however, three patients could eat only soft diet due to loss of teeth. Five patients reported no change of speech after operation, two reported worse speech ability, and one patient reported improved speech after operation. The ipsilateral side of the fibular flap was used when intraoral soft tissue defect with proximal side of the vascular pedicle is required. The contralateral side of the fibular flap was used when extraoral skin defect with proximal side of the vascular pedicle is required.

**Conclusions:**

Osteonecrosis of the jaw is hard to treat because of poor healing process and lack of vascularity. Free fibular flap is the choice of the surgery for jaw bone reconstruction and soft tissue fistula repair. The design and selection of the right or left fibular is dependent on the available vascular pedicle and soft tissue defect sites.

## Background

Osteoradionecrosis (ORN) is a severe complication following radiation therapy of oral cancer, frequently affecting the mandible [1]. It can be defined as a condition in which the irradiated bone becomes exposed through a wound in the overlying mucosa or skin fistula [2]. Marx explains the pathophysiology of ORN using the “3H” principle (hypocellular, hypovascular, and hypoxic tissues) to describe the effect of radiation on the tissue [3]. Schwartz and Kagan [2] reported six characteristics regarding mandibular ORN; first, it is rare when radiation dose is less than 6000 cGy. Second, it is more likely to occur when brachytherapy is used. Third, the mandible must be within the treatment volume to place it at risk. Fourth, the mandible is affected far more frequently than in the maxilla or other bones of the head and neck. Fifth, tooth extraction, minor oral surgery, or trauma can generate the ORN. And sixth, ORN is a problem of impaired wound healing, not an infection but there may be secondary infection.

To treat early stage ORN of the mandible, conservative management (antibiotics, irrigation, and hyperbaric oxygen therapy) may be sufficient [4]. However, patients who present with advanced stage of ORN (ex. orocutaneous fistula, bone destruction at the inferior border of the mandible, pathologic fracture, and severe bone exposure) require more radical treatment such as sequestrectomy, marginal mandibulectomy, or segmental mandibulectomy and reconstruction [5, 6].

A surgical treatment such as block resection or segmental resection generated non-continuous bone defect with unaesthetic and functional problems [7]. These challenges have been met with the development in microvascular tissue transfer techniques, with a variety of free flaps (fibular, ilium, radius, metatarsal, and scapula) available for mandibular reconstruction [8]. For mandible reconstruction, each of these donor sites has significant limitations resulting from either the length of the bone available, the reliability of the associated soft tissue, or donor-site morbidity. The free fibular flap (FFF) is considered as the gold standard to reconstruct the large mandibular bone defect that lead to aesthetic and functional impairment such as swallowing or speech [9, 10]. FFF has many advantages such as consistent shape, sufficient bone and pedicle length, distant location to allow two teams approach, and low donor-site morbidity. Skin islands could be harvested simultaneously for both hard and soft tissue reconstruction.

The microvascular surgery of patients who have received irradiation to the neck is particularly challenging, because of lack of available vessels due to previous neck dissection, skin and mucosa defects, and obliteration of tissue plane. Depending on the site of soft tissue defect (ex. intraoral or extraoral) and site of mandibular bone defect (ex. right or left), the donor site selection of the fibula should be chosen carefully; otherwise, interpositional vein graft is required.

The purpose of this study was to evaluate clinical assessment and quality of life of patients who underwent segmental mandibulectomy and free fibular flap reconstruction due to ORN of the mandible. And we would like to suggest which side of the FFF is favorable for reconstruction without vein graft.

## Methods

This study included eight patients (five males and three females) who visited department of oral and maxillofacial surgery for mandibular reconstruction due to ORN, from March 2008 to December 2015. Institutional review board from our hospital issued an exemption to this study because of the use of collected existing data in such a manner that subjects cannot be identified. Chart review and radiograph data were used for this study. The study was conducted in accordance with the ethical principles provided by the Declaration of Helsinki and the principles of good clinical practice. Patient’s consent forms were obtained before operation. Patients’ demographic data, symptom, primary disease, total radiation dose, flap survival, cause of ORN, and patients’ survival were investigated. Four patients who received radiation in other hospital did not submit the medical record about total radiation dose.

The mean age of eight patients was 60.1 years (range 49–70 years). All patients developed ORN after radiation therapy. Initial symptoms are skin fistula, exposed bone, malocclusion due to pathologic fracture, and pus discharge. The operation was performed under general anesthesia. Selection of the fibular flap was decided by the defect site and skin defect. The ipsilateral side of the fibular flap was used when intraoral soft tissue defect with proximal side of vascular pedicle is required. The contralateral side of the fibular flap was used when extraoral skin defect with proximal side of vascular pedicle is required. Operations were partial mandibulectomy and FFF reconstruction. To stabilize occlusion, 2.0-mm thickness reconstruction plate (Leibinger Co., San Diego, USA) was used for fixation of the mandible with FFF. Feeding vessels were selected from pre-operative angiography.

## Results

The range of total radiation dose of four patients was 56–84 Gy (mean 71 Gy); however, the dose of other four patients was not known. The primary diagnosis of cancer was nasopharyngeal carcinoma (NPC; non-keratinizing carcinoma or undifferentiated carcinoma), OSCC, adenocystic carcinoma (ACC), and mucoepidermoid carcinoma (MEC). The primary tumor site was varied such as nasopharynx, buccal mucosa, and submandibular gland and parotid gland. The mean mandibular defects size was 59.8 mm (range of 38–80 mm). Summary of patients; data are listed in Table 1.

**Table 1:**
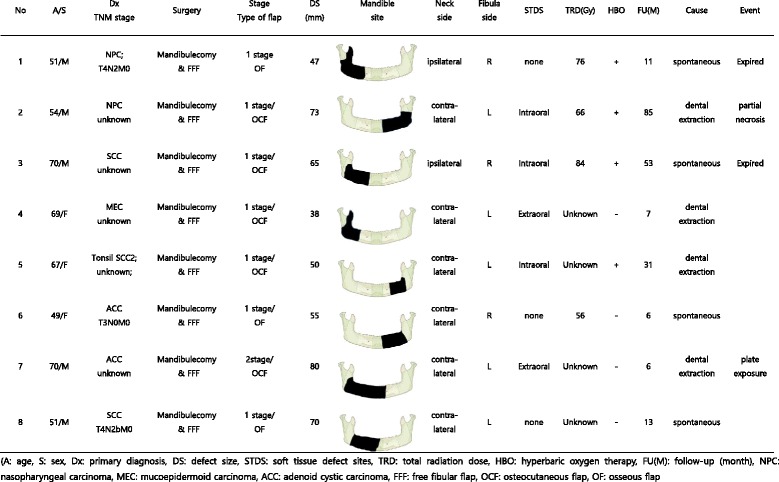
Summary of patients with osteoradionecrosis of the mandible

*A* age, *S* sex, *Dx* primary diagnosis, *DS* defect size, *STDS* soft tissue defect sites, *TRD* total radiation dose, *HBO* hyperbaric oxygen therapy, *FU(M)* follow-up (month), *NPC* nasopharyngeal carcinoma, *MEC* mucoepidermoid carcinoma, *ACC* adenoid cystic carcinoma, *FFF* free fibular flap, *OCF* osteocutaneous flap, *OF* osseous flap

Seven patients were treated with partial mandibulectomy and immediate one-stage reconstruction. One patient (patient no. 7) who had been undergone mandibulectomy and titanium reconstruction plate application in another institution received FFF reconstruction. The reconstruction plate of the no. 7 patient was exposed to the skin.

Patients initially complained with fistulae, followed by pain and difficulty in chewing. Four patients had undergone preoperative hyperbaric oxygen (HBO) therapy which was not effective. The margins of the diseased segments were planned by preoperative panoramic radiographs and CT or MR images. The preoperative imaging is important as a guide, and it can be make a decision of the location of resection. All removed specimens were subjected to histopathological examination to confirm the presence of ORN and to exclude any residual or recurrent tumor.

**Fig. 1:**
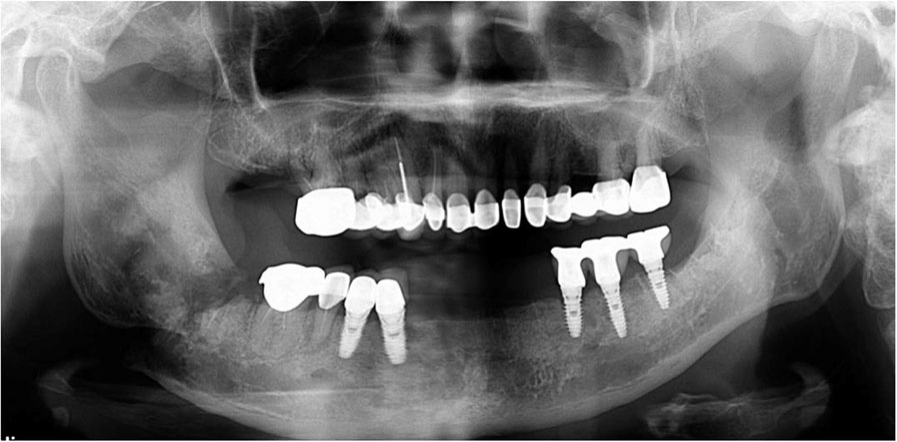
Panoramic radiograph showing pathologic fracture occurred at the right mandibular angle area

**Fig. 2:**
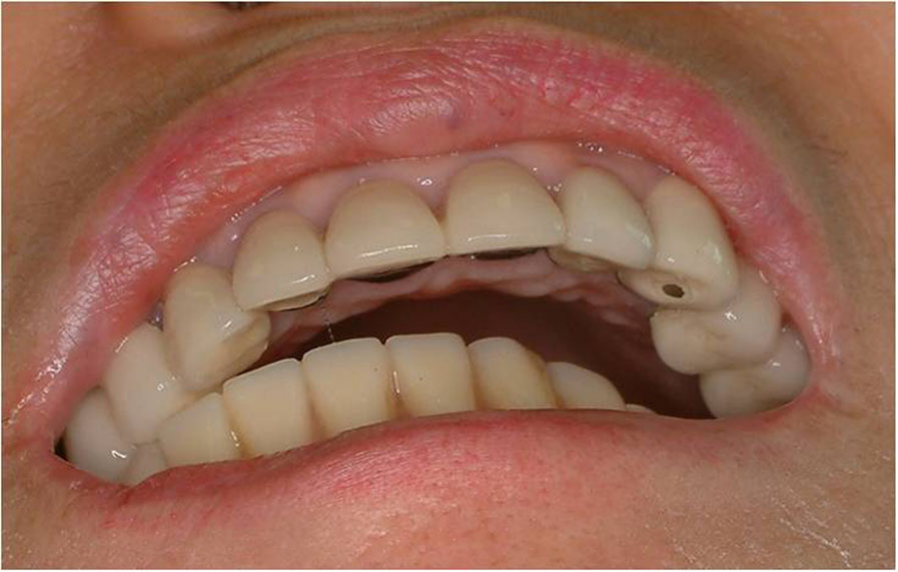
Preoperative intraoral photo (midline deviation and mouth opening limitation)

**Fig. 3:**
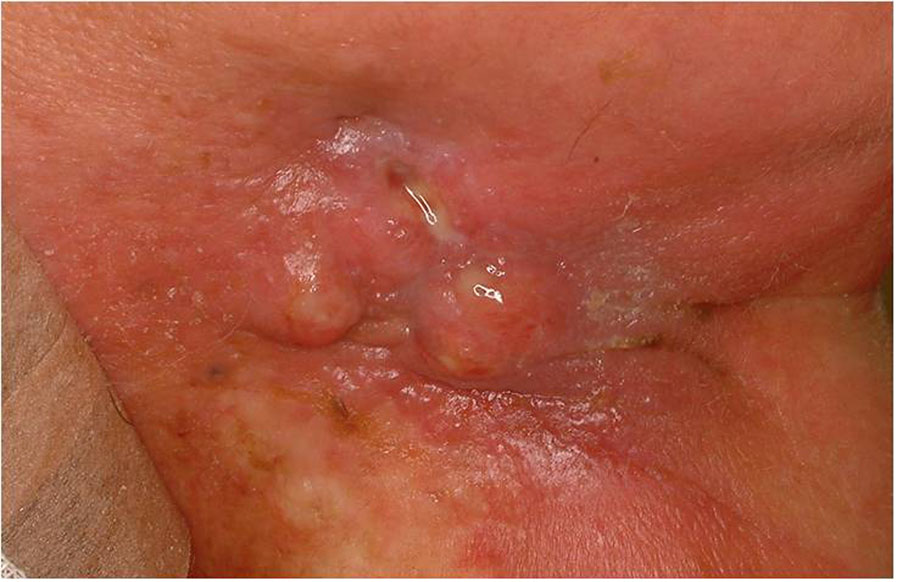
Extraoral fistula with pus discharge

**Fig. 4:**
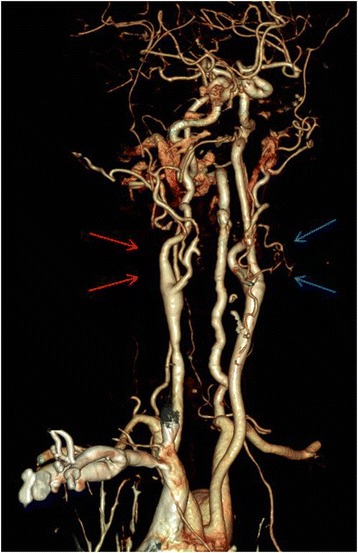
Unavailable vessels at the right neck due to previous neck dissection. (*Red arrow*—no vessels for anastomosis vs. *blue arrow*

**Fig. 5:**
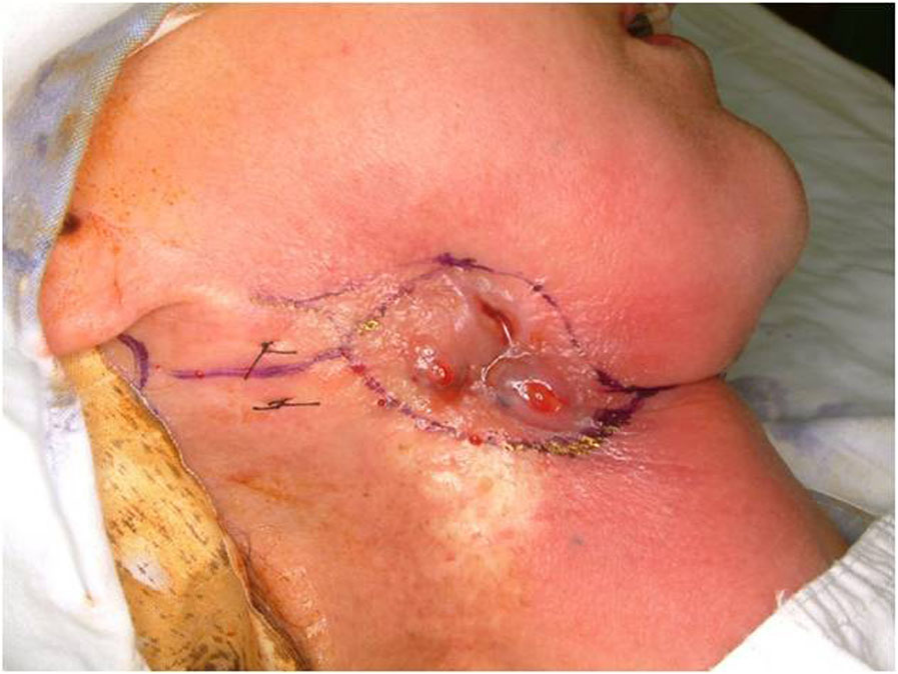
Skin incision design for removal of necrotic skin fistula

**Fig. 6:**
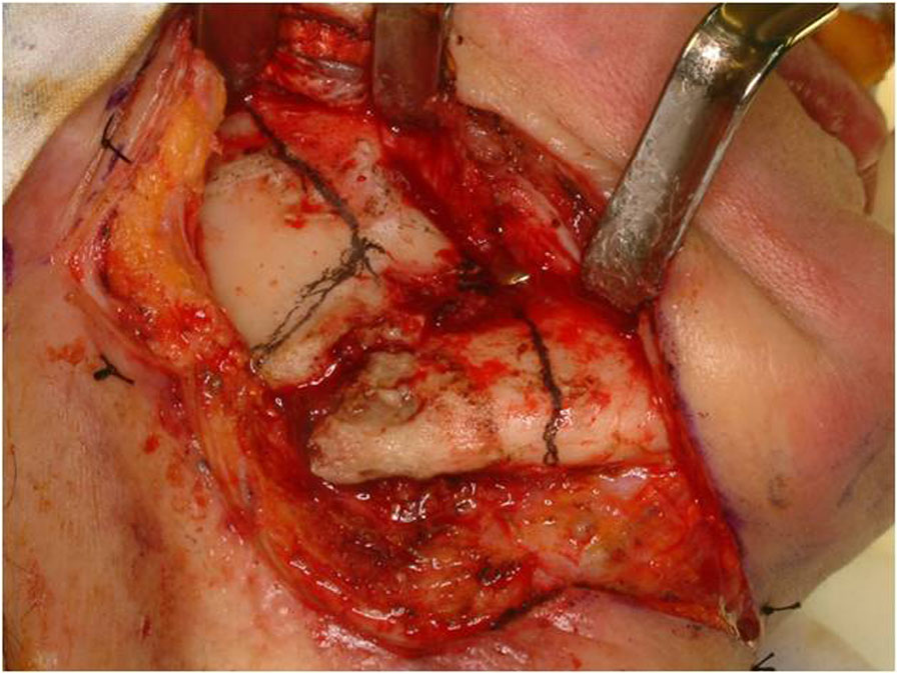
Necrotic bone exposure for mandibulectomy

**Fig. 7:**
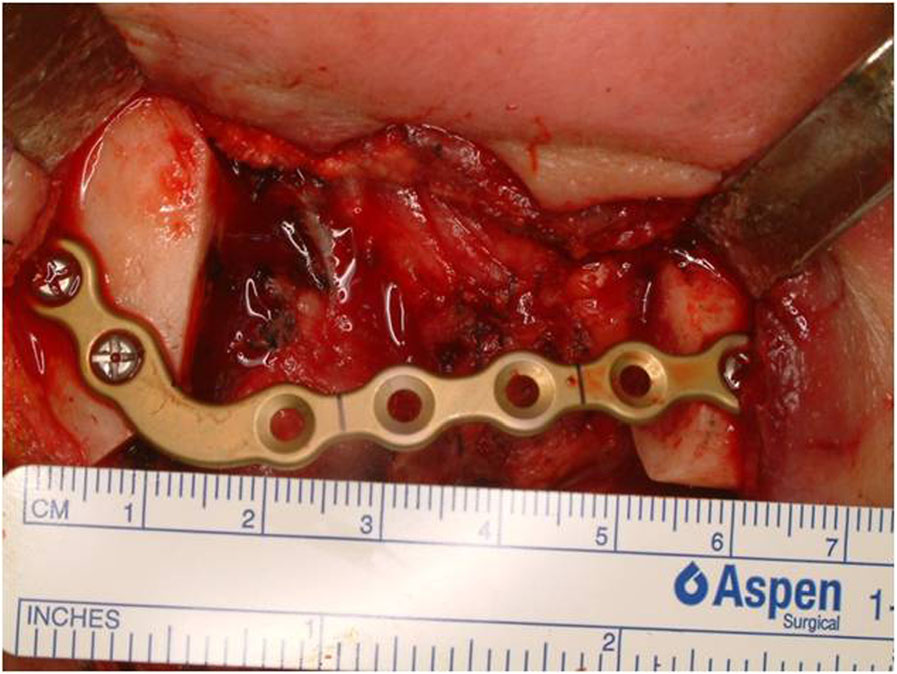
Bone defect about 50 mm. Reconstruction plate application

**Fig. 8:**
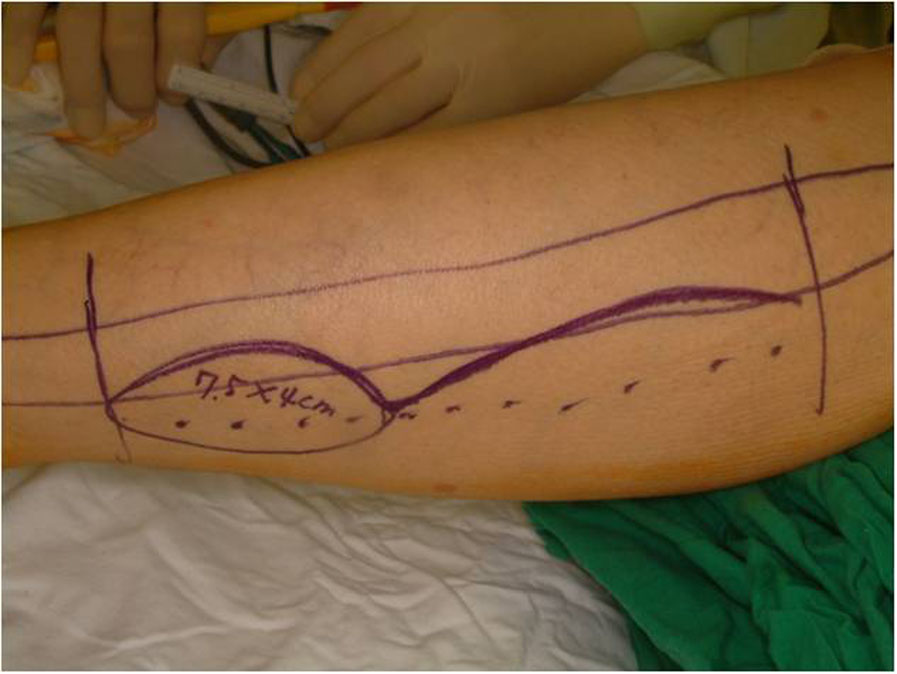
Fibula flap design with skin paddle sized 7.5 × 4 cm

**Fig. 9:**
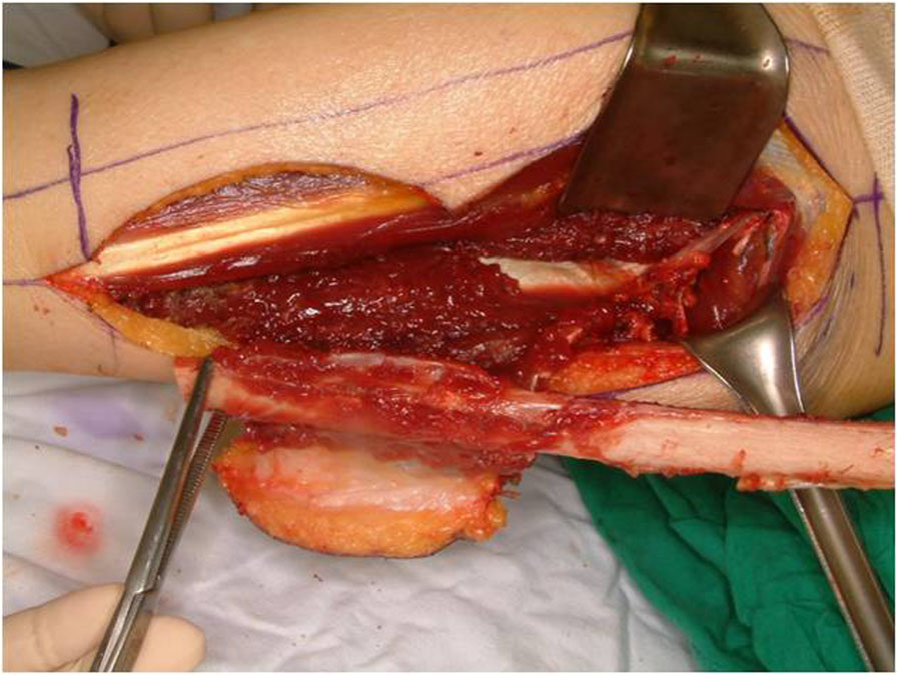
Elevation of osteocutaneous fibula flap

**Fig. 10:**
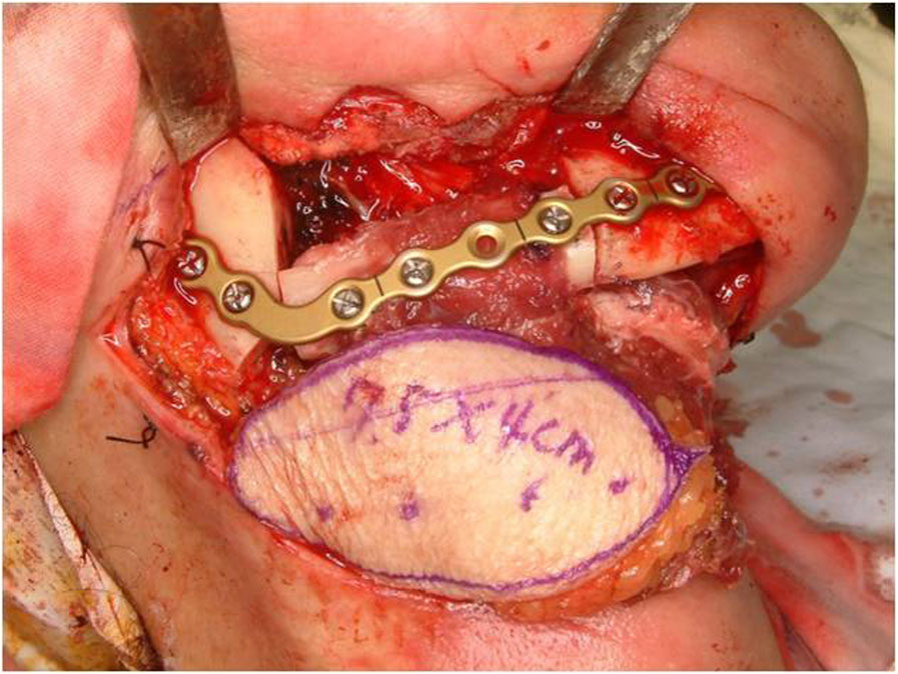
The fibula bone fixed with bicortical screws

**Fig. 11:**
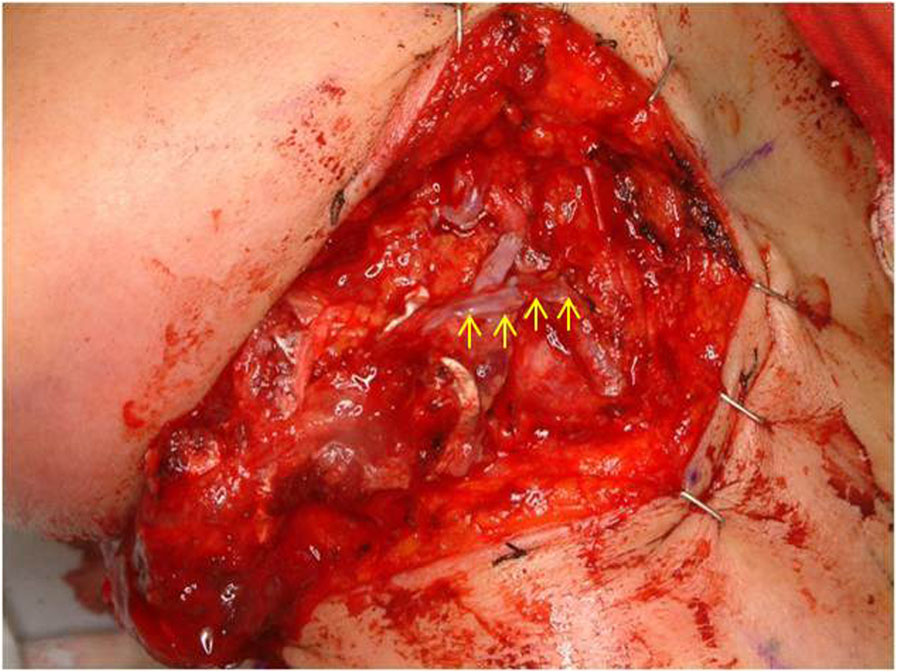
Vessel anastomosis with contralateral facial artery (*yellow arrow*)

**Fig. 12:**
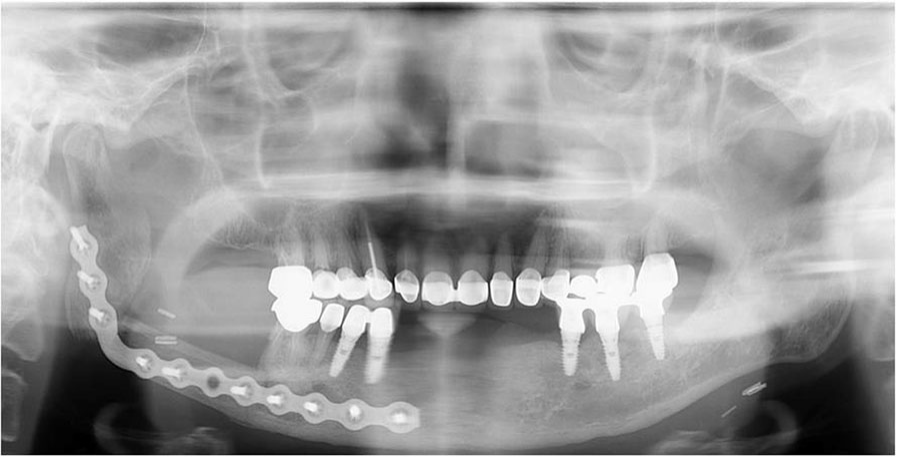
Postoperative panoramic radiograph showing fixation of fibular free flap

**Fig. 13:**
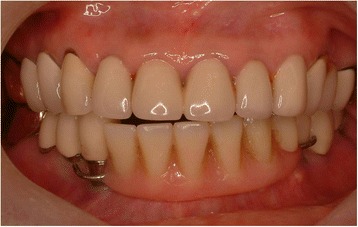
Postoperative intraoral photo showing good occlusion (postoperative 6 months)

**Fig. 14:**
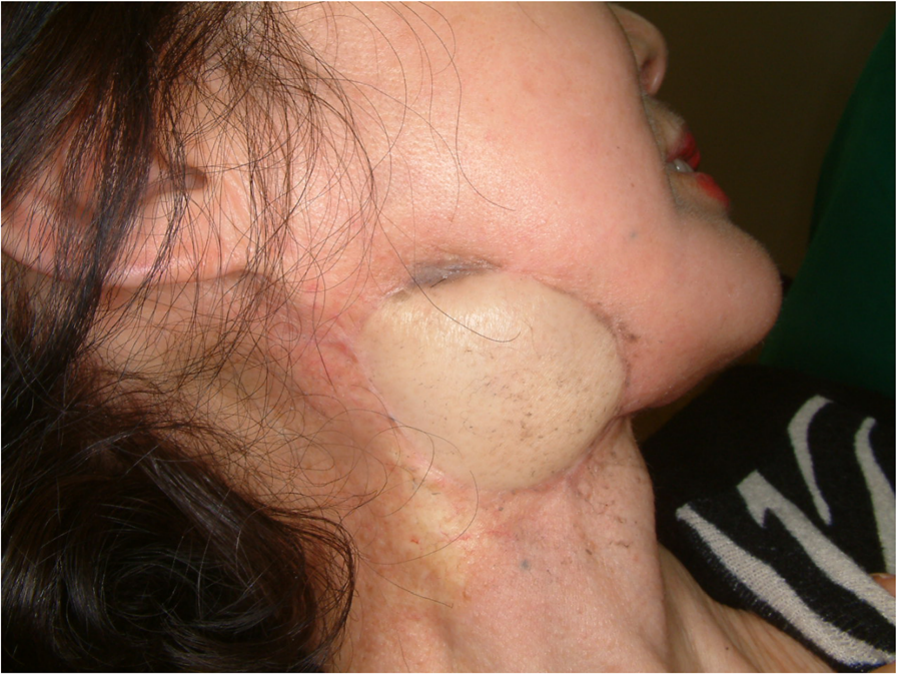
Neck skin photograph showing good healing state (postoperative 6 months)

There was partial skin necrosis in no. 2 patient. The other seven FFFs were successful. Two patients died during follow-up periods due to recurrence of cancer. Primary bone healing was observed in all cases. All the survived patients were relieved of their symptoms and were satisfied with their reconstruction in terms of aesthetic and function. At the last follow-up, six patients were alive (mean follow-up 26.5 months; range 6–85 months). Osseocutaneous flaps were used for reconstruction in five patients, while osseous flaps were used in three patients. Six patients have received neck dissection and irradiation to their ipsilateral neck, vessel anastomosis was performed at the contralateral side without vein graft. Preoperative panoramic radiograph (Fig. 1), initial presentation of occlusion (Fig. 2) and neck skin (Fig. 3), angiography (Fig. 4), operation procedures (Figs. 5, 6, 7, 8, 9, 10, and 11), postoperative panoramic radiograph (Fig. 12), and postoperative occlusion (Fig. 13) and neck skin (Fig. 14) are presented.

## Discussion

Clinically, ORN presents as not healed, exposed bone in a previously irradiated area for at least 2 months unrelated to tumor recurrence [5]. The common signs and symptoms are pain, foul odor, pus drainage, and fistula formation to the mucosa or skin. There have been many studies and classification of ORN. Marx [3] suggested staging system, but the system had a problem that it related to the response to HBO therapy. The staging system of Epstein et al. [11] is an amendment, but also had a problem that was focused on the presence of a pathologic fracture only. Schwartz and Kagan [2] developed a new clinical staging system for ORN of the mandible which is based on clinical experience for 25 years. Stage I is superficial involvement of the mandible and stage II is related to medullary bone necrosis. Stage III is diffuse involvement of the mandible. Division A and B of stage II and III is related to soft tissue ulceration including orocutaneous fistulation. According to the Schwartz and Kagan classification, our patients were all included in stage III division B.

In a series of 80 patients, Thorn et al. [12] found radiation therapy to the floor or mouth or oropharynx placed patients at the greatest risk for development of ORN. By contrast, Notani et al. [13] found in 87 patients with ORN that the most frequent primary tumor site was the tongue. Regardless of the sites, it is certain that the extent of the mandible included in the primary radiation field is a critical factor in determining the potential development of ORN. Tooth extraction in irradiated regions has been recognized as a major risk factor in the development of ORN [14–16]. Beumer et al. [4] noted that ORN associated with post-irradiation extraction required radical mandibular resection in 45 % of patients, as compared with 12 % in ORN associated with pre-irradiation extraction. Meanwhile, ORN can occur in patients who have never taken surgery. In our patients, no. 1 and 2 patients received radiotherapy for their NPC without operation. The other six patients received both surgery and postoperative radiotherapy.

The treatment of ORN is a combination of conservative management and surgical resection. Conservative managements are antibiotics injection, debridement, irrigation, and HBO therapy. HBO has been used widely to ORN patients since 1960s [17]. According to Marx’s theory, HBO can be a good therapy because it can increase oxygen supply in the tissue, stimulating fibroblast proliferation, and angiogenesis [18]. In early stage of ORN, HBO has been used with conservative management to avoid surgical resection of mandible [19, 20]. In a randomized trial by Marx et al. [20], HBO group had a 5.4 % incidence of ORN, as compared with 29.9 % in the penicillin group. However, Annane et al. [21] have reported a less than 5 % incidence of ORN following tooth extraction without HBO therapy. In our patients, four patients received HBO at an initial stage; however, there was no positive effect on ORN. In an advanced ORN (with fistula, pathologic fracture, involvement of inferior border of mandible), the patients require surgical resection with free vascularized bone graft. HBO therapy could not resolve these situations. All the necrotic bone must be removed by surgical resection, and bone margin must be a fresh bone. Gal et al. [22] showed that patients who underwent resection and free osteocutaneous flap reconstruction without HBO had fewer complications than those in whom HBO therapy had been used. In our practice, HBO therapy does not affect surgical result.

The treatment goals of ORN are the alleviation of symptoms and the recovery of aesthetic and function. Especially for patient with mandibular ORN, bone reconstruction is important because of mastication, swallowing, speech, and the harmony of the lower face. In order to achieve optimal surgical result, radical resection and immediate free flap reconstruction have been recommended in the surgical management of mandibular ORN [23, 24]. Placement of a non-viable graft in a radiated area is contraindicated as it is associated with significant complications [25]. Many free-flap donor sites currently exist for mandible reconstruction, but FFF is the first choice option, because the fibula has ample bone length, consistent cross-sectional dimensions, no major variations of bone shape [24, 26]. Most patients required a large bony reconstruction (on average, 6.0 cm), and the complexity of the 3-dimensional bony defect can be restored by the number of osteotomies made to contour the flap adequately.

The surgical treatment of ORN patients is challenging. Several factors contribute to this difficulty. Patients who have received radiation therapy have impaired wound-healing capacity, and some have had previous neck dissection, resulting in the destruction of tissue planes. In a study of carotid-artery images in orthopantograms of 122 patients, Friedlander et al. [27] concluded that patients with total radiation doses sufficient to cause ORN are at a higher risk of developing carotid-artery atherosclerotic lesions than age-matched non-irradiated controls. Careful and delicate dissection and the use of contralateral side vessels for anastomosis can overcome some of these difficulties [28]. Selection of the FFF is important when available vessels are present in the contralateral side. Interposition of vein graft is possible; however, it takes longer time than end-to-end anastomosis at the contralateral side. Our selection criteria are useful, and flaps were all successful when contralateral side anastomosis was performed. Our study involved eight patients with mandibulectomy and fibular reconstruction. Further studies with large number of patients or multicenter study are need.

## Conclusions

Advanced ORN of the mandible should be treated radically with wide resection of necrotic bone and FFF reconstruction. The FFF has many advantages for reconstruction of mandible, especially simultaneous repair of both hard and soft tissues. The design and selection of the right or left fibular are dependent on the available vascular pedicle and soft tissue defect sites.

## References

[1] Store G, Boysen M (2000). Mandibular osteoradionecrosis: clinical behaviour and diagnostic aspects. Clin Otolaryngol Allied Sci.

[2] Schwartz HC, Kagan AR (2002). Osteoradionecrosis of the mandible: scientific basis for clinical staging. Am J Clin Oncol.

[3] Marx RE (1983). A new concept in the treatment of osteoradionecrosis. J Oral Maxillofac Surg.

[4] Beumer J, Harrison R, Sanders B, Kurrasch M (1984). Osteoradionecrosis: predisposing factors and outcomes of therapy. Head Neck Surg.

[5] Teng MS, Futran ND (2005). Osteoradionecrosis of the mandible. Curr Opin Otolaryngol Head Neck Surg.

[6] Costantino PD, Friedman CD, Steinberg MJ (1995). Irradiated bone and its management. Otolaryngol Clin North Am.

[7] Store G, Boysen M, Skjelbred P (2002). Mandibular osteoradionecrosis: reconstructive surgery. Clin Otolaryngol Allied Sci.

[8] Urken ML (1991). Composite free flaps in oromandibular reconstruction. Review of the literature. Arch Otolaryngol Head Neck Surg.

[9] Wilson KM, Rizk NM, Armstrong SL, Gluckman JL (1998). Effects of hemimandibulectomy on quality of life. Laryngoscope.

[10] Schliephake H, Neukam FW, Schmelzeisen R, Varoga B, Schneller H (1995). Long-term quality of life after ablative intraoral tumour surgery. J Craniomaxillofac Surg.

[11] Epstein JB, Wong FL, Stevenson-Moore P (1987). Osteoradionecrosis: clinical experience and a proposal for classification. J Oral Maxillofac Surg.

[12] Thorn JJ, Hansen HS, Specht L, Bastholt L (2000). Osteoradionecrosis of the jaws: clinical characteristics and relation to the field of irradiation. J Oral Maxillofac Surg.

[13] Notani K, Yamazaki Y, Kitada H, Sakakibara N, Fukuda H, Omori K (2003). Management of mandibular osteoradionecrosis corresponding to the severity of osteoradionecrosis and the method of radiotherapy. Head Neck.

[14] Wanifuchi S, Akashi M, Ejima Y, Shinomiya H, Minamikawa T, Furudoi S et al. (2016) Cause and occurrence timing of osteoradionecrosis of the jaw: a retrospective study focusing on prophylactic tooth extraction. Oral Maxillofac Surg. doi:10.1007/s10006-016-0570-5. 10.1007/s10006-016-0570-5 27401528

[15] Kuo TJ, Leung CM, Chang HS, Wu CN, Chen WL, Chen GJ (2016). Jaw osteoradionecrosis and dental extraction after head and neck radiotherapy: a nationwide population-based retrospective study in Taiwan. Oral Oncol.

[16] Nabil S, Samman N (2011). Incidence and prevention of osteoradionecrosis after dental extraction in irradiated patients: a systematic review. Int J Oral Maxillofac Surg.

[17] Pasquier D, Hoelscher T, Schmutz J, Dische S, Mathieu D, Baumann M (2004). Hyperbaric oxygen therapy in the treatment of radio-induced lesions in normal tissues: a literature review. Radiother Oncol.

[18] Jereczek-Fossa BA, Orecchia R (2002). Radiotherapy-induced mandibular bone complications. Cancer Treat Rev.

[19] Chavez JA, Adkinson CD (2001). Adjunctive hyperbaric oxygen in irradiated patients requiring dental extractions: outcomes and complications. J Oral Maxillofac Surg.

[20] Marx RE, Johnson RP, Kline SN (1985). Prevention of osteoradionecrosis: a randomized prospective clinical trial of hyperbaric oxygen versus penicillin. J Am Dent Assoc.

[21] Annane D, Depondt J, Aubert P, Villart M, Gehanno P, Gajdos P (2004). Hyperbaric oxygen therapy for radionecrosis of the jaw: a randomized, placebo-controlled, double-blind trial from the ORN96 study group. J Clin Oncol.

[22] Gal TJ, Yueh B, Futran ND (2003). Influence of prior hyperbaric oxygen therapy in complications following microvascular reconstruction for advanced osteoradionecrosis. Arch Otolaryngol Head Neck Surg.

[23] Chen YB, Chen HC, Hahn LH (1994). Major mandibular reconstruction with vascularized bone grafts: indications and selection of donor tissue. Microsurgery.

[24] Shaha AR, Cordeiro PG, Hidalgo DA, Spiro RH, Strong EW, Zlotolow I (1997). Resection and immediate microvascular reconstruction in the management of osteoradionecrosis of the mandible. Head Neck.

[25] Jisander S, Grenthe B, Salemark L (1999). Treatment of mandibular osteoradionecrosis by cancellous bone grafting. J Oral Maxillofac Surg.

[26] Hidalgo DA (1989). Fibula free flap: a new method of mandible reconstruction. Plast Reconstr Surg.

[27] Friedlander AH, Eichstaedt RM, Friedlander IK, Lambert PM (1998). Detection of radiation-induced, accelerated atherosclerosis in patients with osteoradionecrosis by panoramic radiography. J Oral Maxillofac Surg.

[28] Ang E, Black C, Irish J, Brown DH, Gullane P, O'Sullivan B (2003). Reconstructive options in the treatment of osteoradionecrosis of the craniomaxillofacial skeleton. Br J Plast Surg.

